# Factors influencing adverse events following COVID-19 vaccination

**DOI:** 10.1080/21645515.2024.2323853

**Published:** 2024-03-06

**Authors:** Paola Villanueva, Ellie McDonald, Julio Croda, Mariana Garcia Croda, Margareth Dalcolmo, Glauce dos Santos, Bruno Jardim, Marcus Lacerda, David J. Lynn, Helen Marshall, Roberto D. Oliveira, Jorge Rocha, Alice Sawka, Fernando Val, Laure F. Pittet, Nicole L. Messina, Nigel Curtis

**Affiliations:** a Department of Paediatrics, The University of Melbourne, Parkville, VIC, Australia; b Infection, Immunity & Global Health, Murdoch Children’s Research Institute, Parkville, VIC, Australia; c Infectious Diseases, Royal Children’s Hospital Melbourne, Parkville, VIC, Australia; d Department of General Medicine, Royal Children’s Hospital Melbourne, Parkville, VIC, Australia; e School of Medicine, Federal University of Mato Grosso do Sul, Campo Grande, MS, Brazil; f Fiocruz Mato Grosso do Sul, Fundação Oswaldo Cruz, Campo Grande, Mato Grosso do Sul, Brazil; g Yale School of Public Health, New Haven, CT, USA; h Helio Fraga Reference Center, Oswaldo Cruz Foundation Ministry of Health, Rio de Janeiro, Brazil; i Pontifical Catholic University of Rio de Janeiro, Rio de Janeiro, Brazil; j Fundação de Medicina Tropical Dr. Heitor Vieira Dourado, Manaus, Brazil; k Carlos Borborema Clinical Research Unit, Manaus, Brazil; l Precision Medicine Theme, South Australian Health and Medical Research Institute, Adelaide, SA, Australia; m Flinders Health and Medical Research Institute, Flinders University, Bedford Park, SA, Australia; n Robinson Research Institute and Adelaide Medical School, The University of Adelaide and Department of Paediatrics, Adelaide, SA, Australia; o Nursing Course, State University of Mato Grosso do Sul, Dourados, MS, Brazil; p Graduate Program in Health Sciences, Federal University of Grande Dourados, Dourados, MS, Brazil; q Department of Thoracic Medicine, Royal Adelaide Hospital, Adelaide, SA, Australia; r University of Adelaide Medical School, Adelaide, SA, Australia; s Infectious Diseases Unit, Department of Paediatrics, Gynaecology and Obstetrics, Faculty of Medicine, University of Geneva and University Hospitals of Geneva, Geneva, Switzerland

**Keywords:** COVID-19 vaccine, adverse events, antibody responses

## Abstract

Various novel platform technologies have been used for the development of COVID-19 vaccines. In this nested cohort study among healthcare workers in Australia and Brazil who received three different COVID-19-specific vaccines, we (a) evaluated the incidence of adverse events following immunization (AEFI); (b) compared AEFI by vaccine type, dose and country; (c) identified factors influencing the incidence of AEFI; and (d) assessed the association between reactogenicity and vaccine anti-spike IgG antibody responses. Of 1302 participants who received homologous 2-dose regimens of ChAdOx1-S (Oxford-AstraZeneca), BNT162b2 (Pfizer-BioNTech) or CoronaVac (Sinovac), 1219 (94%) completed vaccine reaction questionnaires. Following the first vaccine dose, the incidence of any systemic reaction was higher in ChAdOx1-S recipients (374/806, 46%) compared with BNT162b2 (55/151, 36%; *p* = 0.02) or CoronaVac (26/262, 10%; *p* < 0.001) recipients. After the second vaccine dose, the incidence of any systemic reaction was higher in BNT162b2 recipients (66/151, 44%) compared with ChAdOx1-S (164/806, 20%; *p* < 0.001) or CoronaVac (23/262, 9%; *p* < 0.001) recipients. AEFI risk was higher in younger participants, females, participants in Australia, and varied by vaccine type and dose. Prior COVID-19 did not impact the risk of AEFI. Participants in Australia compared with Brazil reported a higher incidence of any local reaction (170/231, 74% vs 222/726, 31%, *p* < 0.001) and any systemic reaction (171/231, 74% vs 328/726, 45%, *p* < 0.001), regardless of vaccine type. Following a primary course of ChAdOx1-S or CoronaVac vaccination, participants who did not report AEFI seroconverted at a similar rate to those who reported local or systemic reactions. In conclusion, we found that the incidence of AEFI was influenced by participant age and COVID-19 vaccine type, and differed between participants in Australia and Brazil.

## Introduction

The ChAdOx1-S (Oxford-AstraZeneca), BNT162b2 (Pfizer-BioNTech) and CoronaVac (Sinovac) COVID-19-specific vaccines are among the most frequently used worldwide to protect against coronavirus disease 2019 (COVID-19) caused by severe acute respiratory syndrome coronavirus 2 (SARS-CoV-2).^1,2^

A significant challenge has been vaccine uptake. More than three years after the emergence of COVID-19, nearly one-third of the global population is yet to receive a COVID-19 vaccine dose.^3^ A contributing factor is hesitancy resulting from concerns about the rapid development of vaccines and the potential for adverse events.^4,5^ Population-specific data addressing the risk factors for potential adverse events following immunization (AEFI) can contribute to vaccine acceptance and uptake.^6^

The BRACE (BCG vaccination to reduce the impact of COVID-19 in healthcare workers) trial served as a platform to allow an international study of AEFI, among participants in Australia and Brazil who received intramuscular COVID-19 vaccination (ChAdOx1-S (Oxford-AstraZeneca), BNT162b2 (Pfizer-BioNTech) or CoronaVac (Sinovac)) according to their country-specific vaccine availability. In the first year of the COVID-19 pandemic, a primary series of 2 doses was recommended for ChAdOx1-S (minimum 12-week interval in Australia and Brazil), BNT162b2 (3 and 8 week interval in Australia and Brazil, respectively) and CoronaVac (2 to 4-week interval in Brazil).^7,8^

In this nested, prospective, cohort study among participants who received homologous 2-dose regimens of three different COVID-19-specific vaccines, we aimed to: (a) determine the incidence of AEFI; (b) compare AEFI by vaccine type, dose and country; (c) identify factors influencing the incidence of AEFI; and (d) assess the association between reactogenicity and vaccine antibody responses.

## Materials and methods

### Setting and participants

Participants were healthcare workers recruited in Australia and Brazil between March 2020 and April 2021 in the BRACE trial [NCT04327206] who had COVID-19 vaccine responses solicited. The BRACE trial protocol is detailed elsewhere.^9^ Exclusion criteria in the BRACE trial included previous positive SARS-CoV-2 test or previous receipt of a COVID-19 vaccine. The median interval from blinded BRACE vaccination to the first dose of a COVID-19 vaccine was 94 days (interquartile range 56–156 days).

Participants completed a questionnaire at least one week after each COVID-19 vaccine dose (vaccination administered by their health institution, non-randomized) detailing which vaccine they received and any adverse reactions experienced.

### Data collection

Data were collected using REDCap web application,^10^ including details on demographics and COVID-19 episodes (defined as a symptomatic respiratory or febrile illness with positive COVID-19 PCR or rapid antigen test (RAT)). Information on COVID-19 vaccination (type and timing) and AEFI were collected by telephone interview and web-based questionnaire administered at least seven days after each vaccination dose. The structured questionnaire collected data on local reactions (pain, tenderness, erythema, swelling, itch, regional lymph node swelling), systemic reactions (fever, chills, fatigue, headache, vomiting, diarrhoea, muscle ache, joint ache) and allergic reactions (urticarial, swollen lips, cough/wheeze).

### Sample collection and antibody measurement

For a subset of participants in Brazil, peripheral blood was collected prior to first COVID-19 vaccination and 28 (±2) days following first (ChAdOx1-S) or second (ChAdOx1-S, CoronaVac) COVID-19 vaccine dose, and stored at −80°C (as described previously).^11^ Antibodies against the spike receptor-binding domain of SARS-CoV-2 were measured by chemiluminescent microparticle immunoassays (Abbott, USA), and against nucleocapsid protein (NCP) by Cobas Elecsys anti – SARS-CoV-2 assay (Roche, Switzerland).^12^ Researchers involved in sample processing, selection and testing were blinded to which COVID-19 vaccine participants had received.

### Statistical analysis

The cumulative incidence of local, systemic and allergic reactions in the seven days following first and second COVID-19 vaccine doses was calculated among participants who received the same first two doses of COVID-19 vaccine and who provided vaccine safety data after each dose. Reactions following each vaccination dose were compared between participants using Chi square or Fisher exact tests. Reactions were also compared between participants in Australia and those in Brazil, and between those who received ChAdOx1-S or BNT162b2 vaccines. The severity of local and systemic reactions with the highest incidence after any COVID-19 vaccine dose was graded as per the Food and Drug Administration Toxicity Grading Scale for Healthy Adult and Adolescent Volunteers Enrolled in Preventive Vaccine Clinical Trials.^13^

To identify risk factors for the most common adverse reactions, multivariate logistic regression analysis was applied by administered dose among participants who had negative SARS-CoV-2 serology at recruitment, using age, sex, country, vaccine type and prior COVID-19 as explanatory variables. Odds ratio (OR) and 95% confidence intervals (CI) were determined.

To assess the association between reactogenicity and vaccine antibody responses, linear regression of log-transformed antibody data, adjusted for age, sex, presence of cardiovascular disease, diabetes, chronic respiratory disease, workplace COVID-19 direct contact at baseline, was applied among participants who had negative SARS-CoV-2 serology (spike or NCP) prior to their first COVID-19 vaccine. For antibody concentrations, values below the lower limit of detection were assigned a value of half of the lowest detected value, values above the upper limit of detection were assigned a value of 1.5 times the highest detected value. Adjusted geometric mean antibody ratios were compared between participants with and without local/systemic vaccine reactions. BNT162b2 recipients were excluded from this analysis, due to low number of recipients with antibody data. StataIC 17.0 (Statacorp LP, College Station, TX, USA) was used for analyses.

### Ethics

Ethical approval was obtained from the Royal Children’s Hospital Human Research Ethics Committee (HREC 62586) with subsequent approvals from all participating sites. All research was done in accordance with relevant guidelines and regulations. All participants provided signed informed consent prior to enrollment.

## Results

**Figure 1.**
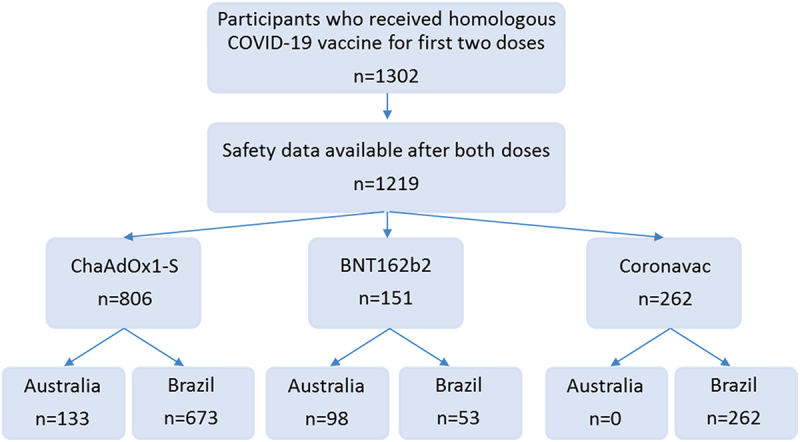
Participants who received homologous COVID-19 vaccine doses.

Of the 1302 participants who received homologous COVID-19 vaccines for the first two doses, 1219 (94%) completed questionnaires providing information on vaccine reactions after both vaccinations (Figure 1). Of these, 988 were in Brazil and 231 in Australia, with an age range of 18 to 73 years (median 41), and the majority (74%) were female. Participants received homologous doses of ChAdOx1-S vaccine (806, 66%; age range 18 to 73 years), BNT162b2 vaccine (151, 12%; 20 to 66 years) or CoronaVac vaccine (262, 21%; 19 to 70 years).

### Incidence of AEFI

**Figure 2.**
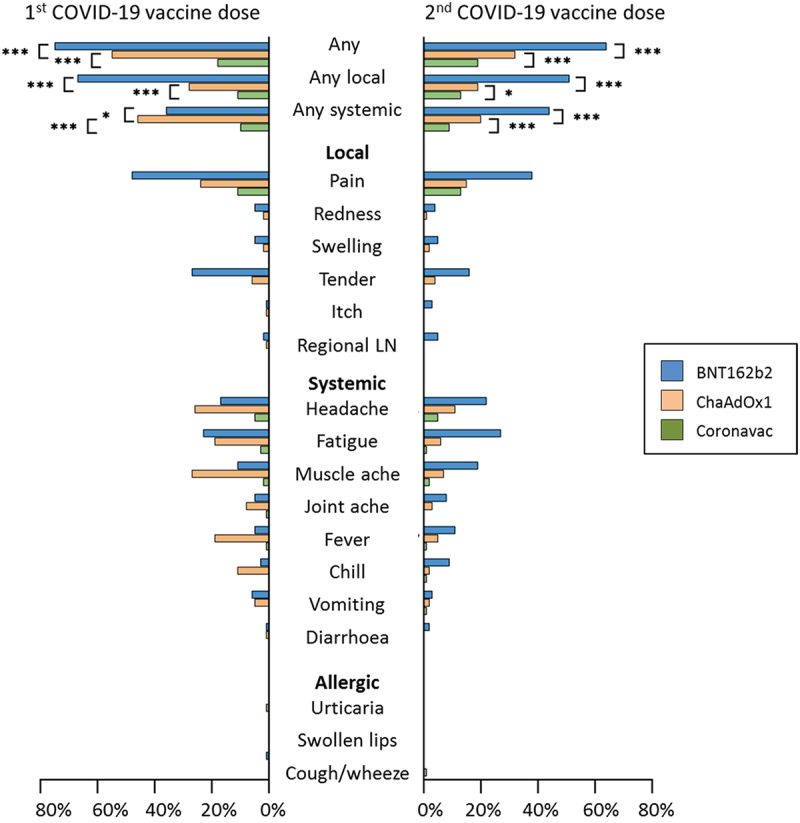
Proportion of participants who reported adverse events in the 7 days following COVID-19 vaccination, by vaccine type and dose.

Abbreviations: LN, lymphadenopathy; *p < 0.05; **p < 0.01; ***p < 0.001.

In the seven days following first or second dose of COVID-19 vaccine, the incidence of any local reaction differed between vaccines (*p* < 0.001), and was higher in BNT162b2 vaccine recipients (111/151, 74%), compared with ChaAdOx1 (281/806, 35%; *p* < 0.001) or CoronaVac (43/262, 16%; *p* < 0.001) vaccine recipients (Figure 2). The local reaction with the highest incidence after any COVID-19 vaccine dose was injection site pain (ChAdOx1-S 242/806, 30%; BNT162b2 90/151, 60%; CoronaVac 43/262, 16%). Injection site pain after any COVID-19 vaccine dose was mainly mild (ChAdOx1-S 222/242, 92%; BNT162b2 79/90, 88%; CoronaVac 43/43, 100%), with a median duration of two days for any COVID-19 vaccine type (Table S1a-b). The systemic reaction with the highest incidence after any COVID-19 vaccine dose was headache (ChAdOx1-S 239/806, 30%; BNT162b2 48/151, 32%; CoronaVac 22/262, 8%). Headache after any COVID-19 vaccine dose was mainly mild (ChAdOx1-S 178/239, 74%; BNT162b2 33/48, 69%; CoronaVac 22/22, 100%), with a median duration of two days for any COVID-19 vaccine type (except following first CoronaVac dose; median headache duration of one day) (Table S1c-d). The frequency of combinations of reactions also varied by vaccine dose (Table S2).

After the first vaccine dose, the incidence of any systemic reaction differed between vaccines (*p* < 0.001), and was higher in ChAdOx1-S recipients (374/806, 46%) compared with BNT162b2 (55/151, 36%; *p* = 0.02) or CoronaVac (26/262, 10%; *p* < 0.001) recipients. After the second vaccine dose, the incidence of any systemic reaction also differed across vaccines (*p* < 0.001), but was higher in BNT162b2 recipients (66/151, 44%) compared with ChAdOx1-S (164/806, 20%; *p* < 0.001) or CoronaVac (23/262, 9%; *p* < 0.001) recipients.

For ChAdOx1-S vaccine recipients, there was a higher incidence of any local reaction and any systemic reaction after the first vaccine dose compared with the second dose (local 225/806, 28% vs. 152/806, 19%, *p* < 0.001; systemic 374/806, 46% vs. 164/806, 20%, *p* < 0.001). For BNT162b2 vaccine recipients, there was a higher incidence of any local reaction after the first vaccine dose compared with the second dose (101/151, 67% vs. 77/151, 51%, *p* < 0.01), and weak evidence for a lower incidence of any systemic reaction after the first vaccine dose compared with the second dose (55/151, 36% vs. 66/151, 44%, *p* = 0.2). For CoronaVac recipients, there was no evidence for a difference in the incidence of any local (28/262, 11% vs. 33/262, 13%, *p* = 0.5) or systemic reaction (26/262, 10% vs. 23/262, 9%, *p* = 0.7) after the first compared with the second vaccine dose.

Allergic reactions were the least reported reactions. After the first dose, the incidence of urticaria was 2/806 (0.2%) in ChAdOx1-S recipients, 2/262 (0.8%) in CoronaVac recipients and 0/151 (0%) in BNT162b2 recipients. After the second dose, the incidence of urticaria was 1/806 (0.1%) in ChAdOx1-S recipients, 1/262 (0.4%) in CoronaVac recipients and 0/151 (0%) in BNT162b2 recipients. The two participants who reported urticaria after the second dose also reported this reaction after the first dose.

**Figure 3.**
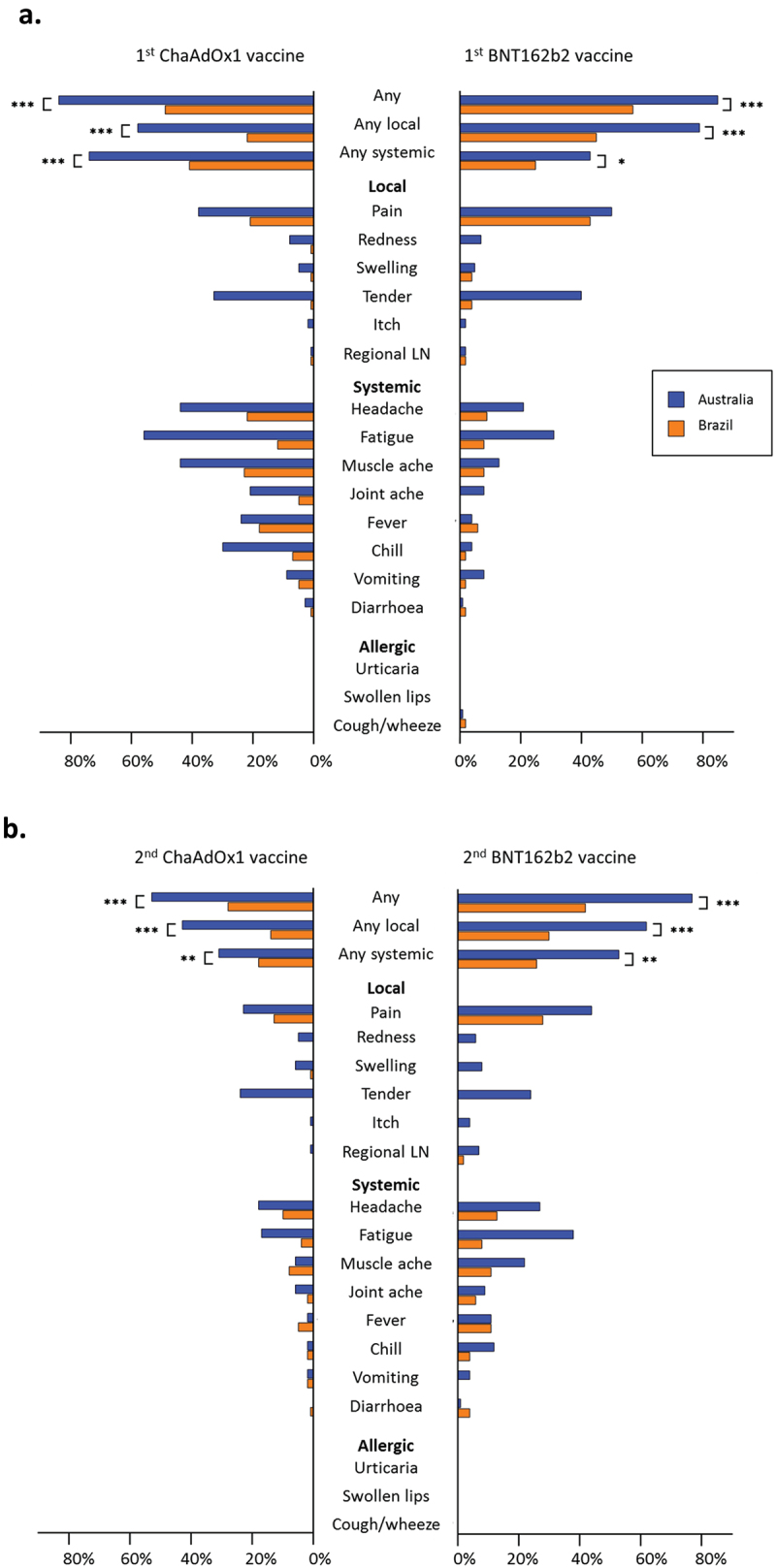
Proportion of participants who reported adverse events in the 7 days following COVID-19 vaccination by country and vaccine type for (a) first vaccine dose and (b) second vaccine dose.

Abbreviations: LN, lymphadenopathy; *p < 0.05; **p < 0.01; ***p < 0.001.

Participants in Australia compared with Brazil (Figure 3) reported a higher incidence of any local reaction (170/231, 74% vs 222/726, 31%, *p* < 0.001) and any systemic reaction (171/231, 74% vs 328/726, 45%, *p* < 0.001), regardless of receiving ChAdOx1-S vaccine or BNT162b2 vaccine, and regardless of dose (first or second).

### Factors associated with any local reaction following COVID-19 vaccination

Following first COVID-19 vaccine dose, in both the univariate and multivariate analysis, any local reaction was more common among younger participants (<50 years old; adjusted OR 2.1, 95%CI 1.4–3.0), participants in Australia (aOR 5.6, 95%CI 3.8–8.2) and varied by COVID-19 vaccine type (BNT162b2 aOR 2.7, 95%CI 1.7–4.4; CoronaVac aOR 0.4, 95%CI 0.2–0.6) (Table 1).

**Table 1.** Demographics and factors investigated for association with local reaction.

			Local reaction after first COVID-19 vaccine dose		Local reaction after second COVID-19 vaccine dose		
	Total vaccinated		Univariate	Multivariate		Univariate	Multivariate
Factor	n = 1040	n (%)	OR (95%CI)	OR (95%CI)	n (%)	OR (95%CI)	OR (95%CI)
Sex							
Male	268	79 (29.5)	1 (reference)	–	46 (17.2)	1 (reference)	–
Female	772	239 (31.0)	1.1 (0.8–1.5), *p* = 0.7		188 (24.4)	1.6 (1.1–2.2), *p* = 0.02	
Age							
≥50 years	283	72 (25.4)	1 (reference)	1 (reference)	51 (18.0)	1 (reference)	1 (reference)
18–49 years	757	246 (32.5)	1.4 (1.0–1.9), *p* = 0.03	2.1 (1.4–3.0), *p* < 0.001	183 (24.2)	1.5 (1.0–2.0), *p* = 0.04	2.1 (1.4–3.1), *p* < 0.001
Country							
Brazil	809	164 (20.3)	1 (reference)	1 (reference)	116 (14.3)	1 (reference)	1 (reference)
Australia	231	154 (66.7)	7.9 (5.7–10.9), *p* < 0.001	5.6 (3.8–8.2), *p* < 0.001	118 (51.1)	6.2 (4.5–8.6), *p* < 0.001	5.4 (3.6–8.0), *p* < 0.001
COVID-19 vaccine							
ChAdOx1-S	713	209 (29.3)	1 (reference)	1 (reference)	142 (19.9)	1 (reference)	1 (reference)
BNT162b2	123	90 (73.2)	6.6 (4.3–10.1), *p* < 0.001	2.7 (1.7–4.4), *p* < 0.001	68 (55.3)	5.0 (3.3–7.4), *p* < 0.001	2.0 (1.2–3.2), *p* < 0.01
CoronaVac	204	19 (9.3)	0.2 (0.2–0.4), *p* < 0.001	0.4 (0.2–0.6), *p* < 0.001	24 (11.8)	0.5 (0.3–0.9), *p* < 0.01	0.8 (0.5–1.4), *p* = 0.5
Prior COVID-19*							
No	974	299 (30.7)	1 (reference)	–	227 (22.7)	1 (reference)	–
Yes	66	19 (28.8)	0.9 (0.5–1.6), *p* = 0.7		7 (18.0)	0.7 (0.3–1.7), *p* = 0.5	

Abbreviations: OR, odds ratio.

*COVID-19 prior to dose (for 2nd dose; between 1st and 2nd dose).

There was no significant association in either analysis with participant sex or prior COVID-19 (Table 1).

Following second COVID-19 vaccine dose, in the univariate analysis, any local reaction was more common among females, younger participants, participants in Australia and those vaccinated with BNT162b2. In the multivariate analysis, any local reaction was more common among younger participants (aOR 2.1, 95%CI 1.4–3.1), participants in Australia (aOR 5.4, 95%CI 3.6–8.0) and those vaccinated with BNT162b2 (aOR 2.0, 95%CI 1.2–3.2) (Table 1).

There was no significant association in either analysis with prior COVID-19 (Table 1).

### Factors associated with any systemic reaction following COVID-19 vaccination

Following first COVID-19 vaccine dose, in both the univariate and multivariate analysis, any systemic reaction was more likely among younger participants (aOR 3.0, 95%CI 2.1–4.3), participants in Australia (aOR 5.3, 95%CI 3.5–8.2) and varied by COVID-19 vaccine type (BNT162b2 aOR 0.2, 95%CI 0.1–0.4; CoronaVac aOR 0.1, 95%CI 0.1–0.2) (Table 2).

**Table 2.** Demographics and factors investigated for association with systemic reaction.

		Systemic reaction after first COVID-19 vaccine dose			Systemic reaction after second COVID-19 vaccine dose		
	Total vaccinated		Univariate	Multivariate		Univariate	Multivariate
TotalFactor	n = 1040	n (%)	OR (95%CI)	OR (95%CI)	n (%)	OR (95%CI)	OR (95%CI)
Sex							
Male	268	92 (34.3)	1 (reference)	–	40 (14.9)	1 (reference)	1 (reference)
Female	772	313 (40.5)	1.3 (1.0–1.7), *p* = 0.1		188 (24.4)	1.8 (1.3–2.7), *p* = 0.001	1.7 (1.2–2.6), *p* < 0.01
Age							
≥50 years	283	77 (27.2)	1 (reference)	1 (reference)	43 (15.2)	1 (reference)	1 (reference)
18–49 years	757	328 (43.3)	2.0 (1.5–2.8), *p* < 0.001	3.0 (2.1–4.3) *p* < 0.001	185 (24.4)	1.8 (1.3–2.6), *p* = 0.001	2.1 (1.4–3.1) *p* < 0.001
Study country							
Brazil	809	264 (32.6)	1 (reference)	1 (reference)	135 (16.7)	1 (reference)	1 (reference)
Australia	231	141 (61.0)	3.2 (2.4–4.4), *p* < 0.001	5.3 (3.5–8.2) *p* < 0.001	93 (40.3)	3.4 (2.4–4.6), *p* < 0.001	2.3 (1.5–3.5) *p* < 0.001
COVID-19 vaccine type							
ChAdOx1-S	713	339 (47.6)	1 (reference)	1 (reference)	149 (20.9)	1 (reference)	1 (reference)
BNT162b2	123	49 (39.8)	0.7 (0.5–1.1), *p* = 0.1	0.2 (0.1–0.4), *p* < 0.001	61 (49.6)	3.7 (2.5–5.5), *p* < 0.001	2.3 (1.4–3.6), *p* = 0.001
CoronaVac	204	17 (8.3)	0.1 (0.1–0.2), *p* < 0.001	0.1 (0.1–0.2), *p* < 0.001	18 (8.8)	0.4 (0.2–0.6), *p* < 0.001	0.4 (0.3–0.8), *p* < 0.01
Prior COVID-19*							
No	974	383 (39.3)	1 (reference)	–	216 (22.2)	1 (reference)	–
Yes	66	22 (33.3)	0.8 (0.5–1.3), *p* = 0.3		12 (18.2)	0.8 (0.3–1.8), *p* = 0.5	

Abbreviations: OR, odds ratio.

*COVID-19 prior to dose (for 2^nd^ dose; between 1^st^ and 2^nd^ dose).

There was no significant association in either analysis with participant sex or prior COVID-19 (Table 2).

Following second COVID-19 vaccine dose, in both the univariate and multivariate analysis, any systemic reaction was more likely among females (aOR 1.7, 95%CI 1.2–2.6), younger participants (aOR 2.1, 95%CI 1.4–3.1), participants in Australia (aOR 2.3, 95%CI 1.5–3.5) and influenced by COVID-19 vaccine type (BNT162b2 aOR 2.3, 95%CI 1.4–3.6; CoronaVac aOR 0.4, 95%CI 0.3–0.8) (Table 2). There was no significant association in either analysis with prior COVID-19 (Table 2).

### Association between reactogenicity and post-vaccine anti-spike IgG level

A subset of 556 participants in Brazil who received a homologous primary COVID-19 vaccination course, completed vaccine reaction questionnaires, and had negative SARS-CoV-2 serology (spike or NCP) prior to their first COVID-19 vaccine dose, also had blood samples available for analysis following first and/or second vaccine doses.

**Figure 4.**
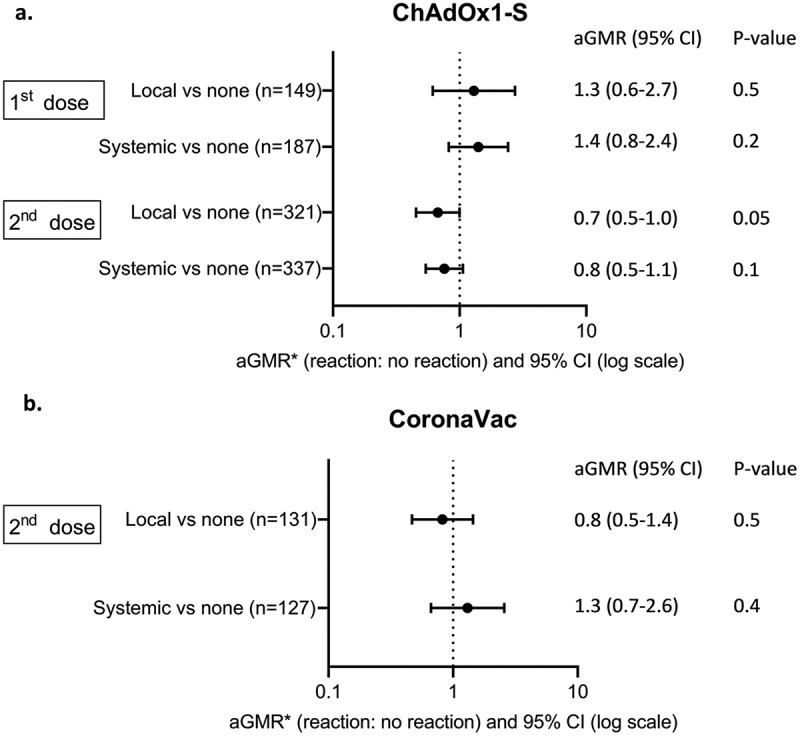
Geometric mean ratio of anti-spike IgG responses by reaction types following COVID-19 vaccination with (a) ChAdOx1-S and (b) CoronaVac.

Among first-dose ChAdOx1-S recipients, there was no association between any local or systemic reaction and anti-spike IgG responses (adjusted geometric mean ratio (aGMR) 1.3, 95%CI 0.6–2.7, *p* = 0.5; aGMR 1.4, 95%CI 0.8–2.4, *p* = 0.2 respectively) (Figure 4a). Among second-dose ChAdOx1-S recipients, there was weak evidence that any local reaction was associated with lower anti-spike IgG responses (aGMR 0.7, 95%CI 0.5–1.0, *p* = 0.05) (Figure 4a). There was no association between any systemic reaction and anti-spike IgG responses (aGMR 0.8, 95%CI 0.5–1.1, *p* = 0.1).

*adjusted for age, sex, presence of cardiovascular disease, diabetes, chronic respiratory disease and workplace COVID-19 contact at baseline.

Among 141 second-dose CoronaVac recipients, there was no association between any local or systemic reaction and anti-spike IgG responses (aGMR 0.8, 95%CI 0.5–1.4, *p* = 0.5; aGMR 1.3, 95%CI 0.7–2.6, *p* = 0.4 respectively) (Figure 4b).

Among either ChAdOx1-S or CoronaVac recipients, there was no difference in the anti-spike IgG seroconversion rate between participants with and without any local or systemic reactions (Table 3).

**Table 3.** Comparison of the proportion of participants with seroconversion in anti-spike IgG following ChAdOx1-S and CoronaVac vaccination, by reaction type.

		Any local reaction N (%)	No reaction N (%)	*p*-value	Any systemic reaction N (%)	No reaction N (%)	*p*-value	Any reaction N (%)	No reaction N (%)	*p*-value
ChAdOx1-S	Seroconversion (1^st^ dose)	31/34 (91%)	111/115 (97%)	0.2	70/72 (97%)	111/115 (97%)	0.6	81/86 (94%)	111/115 (97%)	0.3
	Seroconversion (2^nd^ dose)	51/51 (100%)	268/270 (99%)	0.7	67/67 (100%)	268/270 (99%)	0.6	67/67 (100%)	268/270v(99%)	0.5
CoronaVac	Seroconversion (2^nd^ dose)	16/16 (100%)	115/115 (100%)	–	12/12 (100%)	115/115 (100%)	–	26/26 (100%)	115/115 (100%)	–

## Discussion

In this international study, we found that the reported incidence of adverse reactions following three different COVID-19 vaccines was influenced by both recipient (age, sex) and vaccine (type, dose) factors. In addition, there was a considerable difference between the reported incidence in Australia and Brazil. The most common local (injection pain) and systemic (headache, fatigue, muscle ache) reactions reported were consistent with previous reports.^14–22^

Our finding that the incidence of systemic reactions was higher following first dose ChAdOx1-S compared with BNT162b2 or CoronaVac, and that the incidence of systemic reactions was higher following second dose BNT162b2 compared with ChAdOx1-S or CoronaVac, are consistent with other studies.^18,23–25^ Our study is the first to include a comparison with CoronaVac.

Explanations for the differences in incidence of adverse reactions between countries may include: (i) cultural differences in perception of symptoms following vaccination^26,27^ (ii) the influence of ethnicity on circulating inflammatory cytokine levels^28,29^; and (iii) the phase of the pandemic. At the time of introduction of COVID-19 vaccines, Brazil had one of the highest (and Australia had one of the lowest) burdens of COVID-19 worldwide.^30,31^ In Brazil, the high COVID-19 morbidity and mortality led to considerable psychological and physical stress on healthcare workers,^32^ which may have influenced immunogenicity^33,34^ and/or perception of vaccine reactions. Moreover, the pandemic-associated strain on the health system may have contributed as healthcare workers in Brazil were not as closely monitored (outside of this study) as those in Australia who had to wait fifteen minutes following vaccination. The fervent communication about AEFIs in the Australian media may have also influenced awareness and reporting.

A lower incidence of both local and systemic AEFI among older adults has been observed in previous studies for seasonal influenza vaccine in Australia,^35^ the Netherlands,^36,37^ as well as COVID-19 vaccines (ChAdOx1-S, BNT162b2, mRNA-1273, Ad26.COV2.S) in Germany,^38^ UK,^18,39^ Greece,^39^ the Netherlands^40^ and US.^15^ Explanations for the influence of age on AEFI include immunosenescence^41,42^: older adults produce lower systemic levels of inflammatory cytokines following vaccination.^43^ Another potential contributing factor is a higher tolerance to pain-like symptoms.^44^

Inflammatory responses are important for the development of adaptive immunity following vaccination but are likely also responsible for local and systemic adverse reactions.^44^ The association between vaccine reactogenicity and immunogenicity is gaining increasing research interest. There is some evidence for an association between AEFI and vaccine-induced immune response. A meta-analysis of randomized controlled trials in children showed that prophylactic paracetamol before vaccination (for hepatitis B virus, pneumococcus, *Haemophilus influenzae* type B and poliovirus) was associated with a decreased incidence of local and systemic AEFI, as well as lower antibody responses after vaccination.^45^ In adults, inflammation-related AEFI (local reactions and fever) correlated with higher antibody levels following vaccination with recombinant human papillomavirus or hepatitis E virus vaccines.^46^ The concept that AEFI correlate with a stronger immune response and improved effectiveness is also supported by a study that found that older adults at high risk of cardiovascular disease who experienced mild-moderate AEFI following influenza vaccination, compared with those who did not, were less likely to be hospitalized or die.^47^

Previous studies suggest an association between reactogenicity and antibody responses following a primary course of BNT162b2 vaccination.^48–51^ However, other (smaller) studies of BNT162b2 vaccination have shown inconsistent results. Potential explanations for this include sample size and demographics (age, sex, occupation), measures of reactogenicity (presence or severity of single or group of symptoms), prior COVID-19 exposure,^52^ and the timing and measures of immune response.^12^ The few studies that have investigated other COVID-19 vaccines have found no correlation. However, their findings are limited by small sample size (ChAdOx1-S *n* = 42,^53^ *n* = 340,^51^ *n* = 16^25^; CoronaVac *n* = 53).^54^ Our study, with a larger sample size of participants receiving ChAdOx1-S and CoronaVac vaccination, supports these findings, as there was no strong association between any local or systemic reaction following first or second ChAdOx1-S dose or following second CoronaVac dose, with vaccine antibody response. Importantly, we found that those who did not report AEFI had a similar seroconversion rate to those who reported any local or systemic reaction.

Limitations of our study include that vaccine reactions were self-reported. However, standard questionnaires were used consistent with other studies of reactogenicity. Secondly, participants were healthcare workers, who may have a different perception of adverse reactions than other populations,^55^ and CoronaVac was only administered in Brazil. Thirdly, the cellular immune response was not evaluated. However, anti-spike antibody responses correlate with COVID-19 protection after vaccination.^52,56^

Strengths of our study include prospective recruitment of participants in a large international multicentre study, participants with a known COVID-19 history, the ability to compare different COVID-19 vaccine types within the same study, and adjustment of analyses for known potential confounders.

In conclusion, we found that the incidence of local and systemic reactions following COVID-19 vaccination was influenced by participant age and COVID-19 vaccine type, and differed between participants in Australia and Brazil. Following a primary course of ChAdOx1-S or CoronaVac vaccination, participants without any reactions were as likely to seroconvert as those with local and/or systemic reactions.

## Supplementary Material

Supplementary material3_BRACE trial Consortium Group.pdf

## Data Availability

Deidentified participant data and data dictionary are available to others on request and on completion of a signed data access agreement. Requests can be made in writing to braceresearch@mcri.edu.au.
